# The Role of Car Tyres in the Ecology of Aedes aegypti Mosquitoes in Ghana

**DOI:** 10.21203/rs.3.rs-3286696/v1

**Published:** 2023-08-28

**Authors:** Anisa Abdulai, Christopher Mfum Owusu-Asenso, Christodea Haizel, Sebastian Kow Egyin Mensah, Isaac Kwame Sraku, Abdul Rahim Mohammed, Yaw Akuamoah-Boateng, Akua Obeng Forson, Yaw A. Afrane

**Affiliations:** University of Ghana; University of Ghana; University of Ghana; University of Ghana; University of Ghana; University of Ghana; University of Ghana; University of Ghana; University of Ghana

## Abstract

**Background:**

The *Aedes aegypt*imosquito is an important vector of arboviral diseases including dengue and yellow fever. Despite the wide distribution of the *Aedes aegypti* mosquito, there is limited data on the ecology of *Aedes aegypti* mosquitoes in Ghana. In this study, we report on the oviposition preference and the larval life table of *Aedes aegypti* mosquitoes in Accra, Ghana.

**Methods:**

The oviposition preference of *Aedes*mosquitoes to three habitat types (tyres, drums and bowls) was measured by setting up ovitraps. Ovitraps were checked for the presence of *Aedes* larvae every 3 days. The presence and number of larvae were recorded for each habitat type. Two-hour-old *Aedes aegypti* larvae were introduced into and raised in these three habitat types to undertake larval life tables. The number of surviving larvae at each developmental stage was recorded daily until they emerge as adults.

**Results:**

Car tyres showed a high abundance of *Aedes*larvae (52.33%) than drums (32.49%) and bowls (15.18%) (ANOVA, *F* _ 18.79, *df* _ 2, 159, *P* < 0.001). The mean development time of *Ae. aegypti* larvae was significantly lower in car tyres (7 ± 1 days) compared to that of bowls (9 ± 0.0 days) and drums (12.6 ± 1.5 days) (H (2) = 7.448, *P* = 0.024). The differences in pupation rates and emergence rates were not significant across the habitat types, however, the highest pupation rate was observed in bowls (0.92) and the emergence rate was highest in tyres (0.84). The proportion of first-instar larvae that survived to adults was significantly higher in tyres with a shorter survival time (0.84; 9 days) compared to that of bowls (0.72; 10 days) and drums (0.62 ± 0.2; 13 days) (H (2) = 2.822, *P*= 0.009).

**Conclusion:**

The results confirm that discarded car tyres were the preferred habitat choice for the oviposition of gravid female *Aedes aegypti* mosquitoes and provide the best habitat condition for larval development and survival. These findings are necessary for understanding the ecology of *Aedes* mosquitoes to develop appropriate strategies for their control in Ghana.

## Introduction

Yellow Fever and Dengue fever outbreaks have become more frequent in West Africa in the last 5 years [1–6]. *Aedes aegypti* mosquitoes transmit arboviruses that cause diseases such as Yellow fever, Dengue fever, Zika, Chikungunya and Rift Valley Fever. *Aedes aegypti* is highly anthropophilic and mainly adapted to urban settings [7]. Domestic forms of African *Ae. aegypti* is more adapted to breeding in artificial containers close to human settlements [8,9]. However, the sylvatic forms of *Ae. aegypti* breed in natural breeding habitats such as rock pools, tree holes and fruit husks in forested areas [7]. The main vector control strategies for *Aedes* control are chemical interventions using insecticides and larval source management [10].

The World Health Organization (WHO) recommends using larval source management (LSM) to control the immature stages of *Aedes* mosquitoes [11]. This will help in reducing the densities of *Aedes* vectors within communities. Currently, evidence of insecticide resistance to the four main insecticides in adult *Aedes* mosquitoes has been detected in Ghana and across West Africa [12–16]. Controlling the immature stages of *Aedes* mosquitoes using LSM helps reduce the dependence on the main insecticides by combining Larviciding and habitat modifications and manipulation [17,18].

To control the immature stages of *Aedes* mosquitoes, it is critical to understand the behaviour of these vectors such as their preferred habitat for oviposition and their life history traits. Female *Aedes aegypti* in urban and suburban areas prefer to breed in artificial, man-made containers such as tyres, discarded containers, flower pots or drums [19]. *Aedes aegypti* from different areas have been found to have different oviposition preferences [20–23]. Several studies in West Africa including Ghana have found *Aedes aegypti* mosquitoes breeding predominately in car tyres [15,24–27]. Tyres are especially useful for mosquito reproduction because they are mostly stored outdoors and can collect and maintain rainwater for a long period. Moreover, car tyres serve as an excellent breeding habitat for *Aedes* mosquitoes because decaying leaves from neighbouring trees provide chemical conditions which are similar to tree holes [28]. However, other studies have also found *Aedes* larvae to breed predominately in other habitat types such as large water barrels, animal troughs, plastic containers and septic tanks [28–30].

A female mosquito’s choice of habitat type and oviposition may be influenced by several factors such as the season, habitat size and nutritional availability [19,31]. Female mosquitoes increase the survival and development of their offspring by selecting breeding habitats that reduce the risks of predators and competition [32,33]. For instance, *Aedes* mosquitoes mostly rests and feeds outdoors, so they can explore a wider range of habitat types that are far from human settlements [33]. Several studies in Ghana found *Aedes* mosquito abundance to be significantly higher outdoors than indoors [15,34]. Understanding the oviposition choices of female Aedes mosquitoes will help in designing targeted LSM interventions for these vectors.

Mosquito larval development and survivorship are important determinants of vector densities within an area [35]. Several studies have found that there are variations in life history traits such as larval development and survival in *Ae. aegypti* mosquitoes in different areas [36,37]. This is because different areas may have diverse environmental conditions which may affect larval development, survival and adult emergence [38,39]. Understanding the life history traits of Ghanaian *Aedes aegypti* mosquitoes is very important for the development of appropriate vector control measures for the immature stages of these vectors. This study aims to investigate the oviposition preference and the larval life history traits of *Aedes* mosquitoes in Accra, Ghana. This information will greatly improve vector control strategies especially larval source management for arboviral disease vectors in Ghana.

## Material and Methods

### Study Site

*Aedes* larvae were sampled from different breeding spots within Korle-bu (5° 33’ N 0° 12’ W), Accra, Ghana. Korle-bu is a suburb located in the coastal savannah zone of the Greater Accra Region. This area has the largest teaching and referral hospital in Ghana, thus has an influx of people from different regions across Ghana. This is a populous cosmopolitan area in Accra with numerous vulcanizing spots within the area. The inhabitants also tend to store water in storage containers due to the irregular low of pipe-borne water. There is an abundance of *Aedes* breeding habitats ranging mostly from used car tyres, small containers and water storage drums due to their inadequate water and drainage systems.

### *Aedes* Oviposition in Three Habitat Types

An oviposition experiment was set up to investigate the preferred habitat type for *Aedes* mosquitoes using three habitat types, i.e., car tyres, bowls and drums. Car tyre ovitraps were made by cutting a car tyre into three parts that can hold water. Each car tyres ovitrap was approximately 56 cm in length and 15cm in height with an aperture of 5cm. Bowls were plastic, black and had a capacity of 5 litres water capacity. Drums had a water capacity of 50 litres and were made of plastic. At a particular spot, all three different habit types were set up to determine, which type was preferred for oviposition by gravid female *Aedes* mosquitoes.

Each container type was filled with 2 to 5 litres of rainwater and was placed in the natural environment close to where other habitats have been found in the past. For the reproducibility of the experiment, three replicates of each habitat type were set up in different areas. Larval abundance was used as a proxy for oviposition preference because of the difficulty in identifying and counting *Aedes* eggs that were laid in the different habitats especially tyres because of their dark surface. The replicates were checked for the presence of larvae every three days. The number of larvae were recorded for each habitat type. Larvae were removed and the water was replaced after every recording.

### Larval life table in three habitat types

A life table experiments were performed to determine the life history traits of *Aedes aegypti* mosquitoes in different habitat types. The life-table components investigated were larval development time, pupation rates, emergence rate and sex ratio. Experiments were set up in three replicates each, for three different habitat types, i.e., car tyres, drums and bowls. For each replicate, thirty 2-hour-old larvae were introduced into each habitat type, containing 2 to 5 litres of rainwater and covered with a muslin net. Each habitat type was placed in its natural environment. The replicates were observed daily and the volume of water was replenished daily due to evaporation.

Developmental stages of surviving *Ae. aegypti* mosquito larvae were assessed daily; alive and dead larvae were recorded. Pupae collected from the habitat types were held in pre-labelled individual paper cups with water of a depth of 25 ml for adult emergence. The paper cups containing one pupa per cup were covered with muslin netting and observed daily for adult emergence. Pupae mortality, adult mosquitoes that emerged (emergence rate), and the sexes were recorded daily. When pupation started, habitats were visited twice a day, at 8 am and 5 pm daily for pupae collections.

### Data Analysis

Larval abundance was calculated as the percentage of number of larvae obtained per each habitat type over the total number of larvae collected. This was used to measure the oviposition preference of *Aedes* mosquitoes to the three habitat types. For the larval life table experiment, the larval development time was recorded in days as the duration from the first instar larval stage to the pupal stage. Mean larval development time was defined as the average duration, in days, of first-instar larvae to develop into pupae. The pupation rate was calculated as the sum of the total number of pupae per the sum of the total number of 1^st^ Instar larvae. The emergence rate was calculated as the sum of the number of adults that emerged per the total number of pupae.

Survivorship of *Ae. aegypti* larvae was calculated as the proportion of first-instar larvae that survived to adults. The ratio of females to males was determined by counting and recording the number of males and females that emerge per day over the total number of adults that emerged. The ANOVA test and Kruskal-Wallis ranked sum test was used to test the statistical significance of the different habitat types on larval survivorship and larval development time wherever appropriate. Dunn’s test was used to compare the significance of the means. Pearson Chi-square test was used to test for significance in survivorship. All data analyses were performed using R version 3.6.3.

## Results

### Aedes immature abundance in three different habitat types

**Figure 1:**
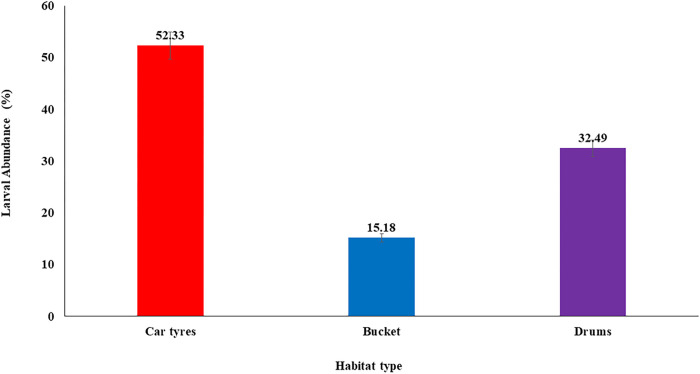
Abundance of immature *Aedes* mosquitoes collected from different habitat types (tyres, bowls and drums). Error bars represent the 95% confidence interval of the mean.

A total of 4,059 immature *Aedes* mosquitoes were collected during the entire sampling period. The highest numbers of *Aedes* immatures were found in used tyres (2124/ 4059; 52.33%) as compared to that of drums (1319/4059; 32.49%). Bowls recorded the least abundance of *Aedes* immatures (616/4059; 15.18%) (Fig 1). There was a significant difference in the abundance of immature in the three habitat types (ANOVA, *F* _ 18.79, *df* _ 2, 159, *P* < 0.001).

### Development Time and Survival of Immature Stages of *Aedes aegypti*

The mean larval to pupae development time for immature *Aedes aegypti* mosquitoes was lower in tyres, 7 days as compared to bowls (9 days) and drums (12.7 days) (H (2) = 7.448, *P* = 0.024). A pairwise post-hoc Dunn test with Bonferroni adjustments showed that there was a significant difference in the mean larval to pupae development time between tyres and drums (*P* = 0.009) but no significant difference between tyres and bowls (*P* = 0.258). Furthermore, there was no significant difference in the mean larval to adult development time in males from the three habitat types (H (2) = 5.728, *P*= 0.06). However, there was a significant difference in the mean larval to adult development time of female *Ae. aegypti* mosquitoes (H (2) = 6.054, *P*= 0.048).

### Pupation rate, emergence and survivorship of Immature *Aedes aegypti* in the different habitat types

A higher proportion of larvae pupated in bowls, (0.92) compared to that of tyres (0.88) and drums (0.75) but the observed differences were not significant (H (2) = 2.667, *df* = 2, *P*= 0.263). Out of the proportion of larvae that pupated, those that emerged as adults were 0.84 in tyres, 0.8 in drums and 0.77 in bowls (ANOVA, *F* _ 0.38, *df* _ 2, 6, *P* < 0.697). The proportion of first-instar larvae that survived to adults was greater in tyres (0.84 ± 0.1) than that in bowls, 0.72 ± 0.2 and drums, 0.62 ± 0.2. However, the differences in survivorship were not significant across the three habitat types (H (2) =2.822, *P*= 0.238). This is shown in Table 2.

**Figure 2:**
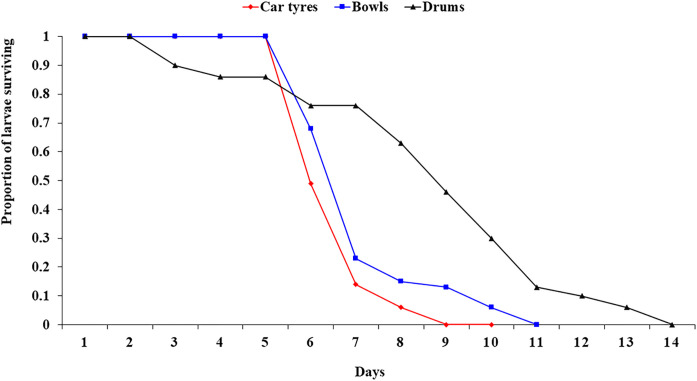
*Aedes aegypti* larval survivorship curve across the different habitat types

*Ae. aegypti* larvae from car tyres showed a significantly shorter survival length of 9 days compared to that of drums (13 days) (H (2) = 2.822, *P*= 0.009). Larval survivorship reached 0% (all larvae became pupa) after 10 days in bowls (Figure 2).

### Sex Ratio of immature *Aedes aegypti* mosquitoes in different habitat types

The proportion of males that emerged was higher in tyres (65.79%) and bowls (60%) compared to that of males that emerged from drums (37.5%). The proportion of emerged females was higher in drums (62.5%) compared to that of bowls (40%) and drums (34.21%). There was a significant association between habitat type and the sex of emerged adults, (*χ*2 = 11.0601, *df* = 2, *P*= 0.004) (Table 3).

## Discussion

Understanding the ecology and biology of *Aedes* mosquitoes is crucial for the control of Aedes-borne diseases. Development times and survivorship of various stages of mosquitoes under different environments are of particular importance, as they affect the vectorial capacity, which is tightly linked to mosquito-borne disease transmission [40]. This study provides evidence of the oviposition preferences and larval life history traits of *Aedes aegypti* mosquitoes in different habitat types in Accra, Ghana. Findings from our study showed high Aedes larval abundance in tyres compared to that of the other habitat types. *Aedes aegypti* larvae from tyres showed a significantly shorter development time and high survivorship compared to the other habitat types.

*Aedes* immature abundance was used as a proxy for oviposition preference in three different habitat types. The highest larval abundance was observed in tyres, suggesting that Ghanaian *Aedes* mosquitoes prefer to breed in tyres compared to other habitat types. This finding is in line with that of Owusu-Asenso et al (2022) where car tyres produced the highest densities of *Aedes* larvae [15]. Car tyres are among the most productive aquatic habitats across West Africa [20, 26, 41]. This may be because discarded car tyres are less prone to disturbance as compared to other habitat types such as containers, tin cans or coconut shells. Also, the internal condition in car tyres such as reduced light and low humidity attract gravid female *Aedes* mosquitoes [42]. Car tyres have a narrow opening thus providing some level of shade to the immature larvae and reducing the amount of light entering the habitat [20]. High mosquito densities have been associated with habitats that have some level of shading [43].

Thus, car tyres may be targeted for larval source management in the control of *Aedes* mosquitoes. Although car tyres are the most productive habitat type, discarded containers are also a common source of aquatic habitats. Drums were the second most abundant habitat in our study [20]. A risk factor for the presence of *Aedes* mosquitoes is the storage of water in drums for drinking or domestic use. A study from Cape Coast, Ghana found more storage containers to be infested with *Aedes* larvae in areas with water storage compared to areas with adequate access to piped water [25].

This study observed that the mean development time was significantly longer in the *Aedes aegypti* larvae from drums compared to Aedes larvae in tyres. The mean development time was similar between tyres and bowls and shorter as compared to that of drums. The short mean development time of *Aedes* mosquito larvae has important epidemiological implications. Rapid larval development favors higher vector densities, which may increase disease transmission. This is because the longer development time will expose the larvae to predation and loss of habitat through desiccation [20]. This negatively impacts the vectorial capacity of the vectors [44].

There were no significant differences in the emergence of *Ae. aegypti* mosquitoes across the three habitat types. However, the proportion of emerged adults was higher in car tyres and drums compared to bowls. Larval survivorship was higher in car tyres coupled by high pupation and emergence rates. Compared to other habitat types, *Ae. Aegypti* larvae from car tyres showed a significantly short survival length. These findings suggest that car tyres may be responsible for the high densities of adult *Aedes* mosquitoes, which may facilitate arboviral transmission in Accra, Ghana. The experiments were conducted in controlled environment thus this study did not take into account potential biological factors such as predators and competition, which may affect may survivorship. Furthermore, temperature and relative humidity was not measured, thus, its effect on the abundance of *Aedes* larvae was not determined.

## Conclusion

This study showed that car tyres are the preferred choice for oviposition for gravid female *Aedes* mosquitoes. Furthermore, this study provides the first report on the life history parameters of *Aedes aegypti* mosquitoes from Korle-Bu, Accra, Ghana. *Ae. aegypti* larvae from car tyres showed a shorter larval development time and higher survivorship compared to other habitat types. This suggests that tyres may be playing a significant role in the ecology of *Aedes* mosquitoes, thus facilitating arboviral transmission. This study provides baseline information that is essential for wider studies towards a better understanding of the ecology of *Aedes* mosquitoes to develop appropriate strategies for their control in Ghana.

## Figures and Tables

**Table 1:** Larval development time of immature *Aedes aegypti* mosquitoes in different habitat types

Habitat Type	Mean larval-pupae development time	Mean development time of males (days)	Mean development time of females (days)
Tyres	7 ± 1^a^	8 ± 1^a^	8.6 ± 0.5^a^
Bowls	9 ± 0.0^a^	8.3 ± 0.5^a^	8.3 ± 0.5^a^
Drums	12.6 ± 1.5^b^	12 ± 1^b^	14.3 ± 1.1^b^

Values are means ± standard deviations. The letter symbol following the numerical values indicates the results of multiple comparison tests, and values with the same letter in each column were not statistically significant at *P*<0.05 and those with different letters in each column were statistically significant at *P*<0.05.

**Table 2:** Pupation rate and emergence rate of immature *Aedes aegypti* mosquitoes in different habitat types

Habitat Type	Pupation (x ± SD)	Emergence (x ± SD)	Survivorship (x ± SD)
Tyres	0.88 ± 0.02	0.84 ± 0.1	0.84 ± 0.1^a^
Bowls	0.92 ± 0.17	0.77 ± 0.7	0.72 ± 0.2^a^
Drums	0.75 ± 0.22	0.8 ± 0.12	0.62 ± 0.2^b^

Values are means ± standard deviations. The differences in the proportion of larvae that pupated and emerged between the three habitat types, tyres, bowls and drums were not statistically significant (P>0.05).

**Table 3:** The number of males and females that emerged from the different habitat types

Habitat type	Sample Size	Male %	Female %	Sex Ratio (Female: Male)
Tyres	76	65.79	34.21	1:1.8^a^
Bowls	55	60	40	1:1.9^a^
Drums	56	37.5	62.5	1:0.6^b^

Values are means percentages. The letters symbol following the numerical values indicate the results of multiple comparison tests, and values with the same letter in each column were not statistically significant at *P*<0.05 and those with different letters in each column were statistically significant at *P*<0.05.

## Data Availability

All datasets generated and/or analysed during this study are available on request.
